# Matter Over Mind: A Randomised-Controlled Trial of Single-Session Biofeedback Training on Performance Anxiety and Heart Rate Variability in Musicians

**DOI:** 10.1371/journal.pone.0046597

**Published:** 2012-10-04

**Authors:** Ruth Wells, Tim Outhred, James A. J. Heathers, Daniel S. Quintana, Andrew H. Kemp

**Affiliations:** SCAN Research & Teaching Unit, School of Psychology, University of Sydney, Sydney, Australia; Institute of Psychiatry at the Federal University of Rio de Janeiro, Brazil

## Abstract

**Background:**

Musical performance is a skilled activity performed under intense pressure, thus is often a profound source of anxiety. In other contexts, anxiety and its concomitant symptoms of sympathetic nervous system arousal have been successfully ameliorated with HRV biofeedback (HRV BF), a technique involving slow breathing which augments autonomic and emotional regulatory capacity. *Objective:* This randomised-controlled study explored the impact of a single 30-minute session of HRV BF on anxiety in response to a highly stressful music performance.

**Methods:**

A total of 46 trained musicians participated in this study and were randomly allocated to a slow breathing with or without biofeedback or no-treatment control group. A 3 Group×2 Time mixed experimental design was employed to compare the effect of group before and after intervention on performance anxiety (STAI-S) and frequency domain measures of HRV.

**Results:**

Slow breathing groups (n = 30) showed significantly greater improvements in high frequency (HF) and LF/HF ratio measures of HRV relative to control (n = 15) during 5 minute recordings of performance anticipation following the intervention (effect size: η^2^ = 0.122 and η^2^ = 0.116, respectively). The addition of biofeedback to a slow breathing protocol did not produce differential results. While intervention groups did not exhibit an overall reduction in self-reported anxiety, participants with high baseline anxiety who received the intervention (n = 15) displayed greater reductions in self-reported state anxiety relative to those in the control condition (n = 7) (*r = *0.379).

**Conclusions:**

These findings indicate that a single session of slow breathing, regardless of biofeedback, is sufficient for controlling physiological arousal in anticipation of psychosocial stress associated with music performance and that slow breathing is particularly helpful for musicians with high levels of anxiety. Future research is needed to further examine the effects of HRV BF as a low-cost, non-pharmacological treatment for music performance anxiety.

## Introduction

The work environment of professional musicians can be a stressful world of extreme highs and lows [1], [2] often leading to pre-performance anxiety [3] and post-performance depression [2], [4]. Performance anxiety (PA) has a disproportionate effect on musicians [5], with estimates of prevalence ranging from 15% to 59% [6]–[9]. Physiological symptoms such as tremors, raised heart rate (HR) [10], dry mouth and hyperventilation [11] and cognitive symptoms, including an inability to concentrate [3], interfere with a musician’s ability to perform to the highest possible standard [10] precisely when they need it the most [3]. Musicians work in a highly competitive environment, where negative evaluation by peers, reviewers and audience members is part of the vocation [12]. Inability to perform due to anxiety can lead to missed opportunities and loss of income. Given these factors, it is not surprising that a large number of performing musicians rely on pharmacological aids (such as beta-adrenergic antagonists), often without prescription [6] which can lead to adverse mood states [13], [14]. Others turn to unhelpful coping strategies, such as alcohol consumption [15], [16], in order to minimise the physiological manifestations of PA during performance [6].

Heart rate variability biofeedback (HRV BF) is an intervention involving slow breathing (6 breathes per minute), which dampens physiological arousal in stressful situations [17]. It has been associated with reductions in anxiety symptoms [18], [19] and improved cognitive performance [20]. Heart Rate Variability (HRV) is an index of beat to beat changes in heart rate and is a psychophysiological marker for physical and mental health [21]–[23]. Low HRV is associated with mood and anxiety disorders [24]–[27]. High HRV has a protective effect and is associated with good health [28] and well-being [29].

High frequency (HF) HRV reflects the magnitude of PNS influence on HR associated with breathing – respiratory sinus arrhythmia (RSA) [30] – which is carried to the heart via the tenth cranial (vagus) nerve. Power spectral analysis partitions the observed variability into components of high (HF), low (LF) and very low frequency (VLF) using spectral decomposition, most commonly a Fast Fourier Transformation [30]. PNS, sympathetic nervous system (SNS) and blood pressure mechanisms operate on different time scales, so each of these components is thought to be associated with different sources of variability [31]. The HF component (between 0.15–0.4 Hz) is known to be PNS mediated [32]–[34], while the LF component (0.04–0.15 Hz) reflects a combination of PNS and SNS activity [35] via baroreflex function [31]. As a consequence, the LF/HF ratio is thought to provide information about the relationship of vagal input to the other sources of variability [36].

Higher levels of resting state HF HRV may indicate increased ability to inhibit SNS-mediated arousal [37], leading to increased behavioural flexibility [38] in the face of stress and reduced anxiety, as argued by Thayer and colleagues [39]–[41]. Maladaptive emotion regulation strategies, such as worry, lead to acute reductions in HRV [25] whereas social approach behaviors are associated with increased parasympathetic activity, which is facilitated by an increase in vagal tone [42]. Porges [21] argues that human social engagement is enabled by a functional network of facial, vocal and cardiac cranial nerves, including the vagus. Inhibition of threat-related systems associated with anxiety is dependent upon fast-acting, bi-directional communication between visceral organs and brainstem nuclei via the vagus nerve. Rapid modulations of arousal through vagal mechanisms support adaptive emotion regulation by helping adjust emotions to their context [22]. Anxiety is related to an individual’s ability to employ appropriate emotion regulation strategies [43]–[46], thus, improving HRV levels may help to alleviate anxiety.

Physiological processes, such as breathing and HR, are directly involved in emotion regulation during music performance. Disruption to habitual breathing patterns appear to have a bidirectional relationship with anxiety [47]. Studer et al. [11] found that highly anxious individuals reported more hyperventilation prior to music performance. In contrast, slow, deep breathing can have remarkably beneficial effects, lowering blood pressure [48], increasing blood-oxygen concentration and perhaps altering baroreflex function [49], [50]. Slow breathing causes large increases in RSA amplitude [51]. Bernardi et al. [52] found slow breathing in simulated altitude reduced SNS activation under hypoxic stress and that those who regularly practice slow yogic breathing displayed a greater SNS inhibition under stress. Interestingly, slow breathing is found in disparate meditative traditions [53]. It should be noted here that while clinically intriguing, the precise autonomic and regulatory mechanisms provoked by slow breathing are poorly understood. While there is undoubtedly a baroreflex component to 0.1 Hz breathing, baroreflex sensitivity does not show immediate provocation to the modified Oxford method of cardiovagal baroreflex assessment [54].

The aim of HRV BF is to slow the breathing (normally ∼15 breaths per minute) to coincide with HRV changes due to the baroreflex (∼6 cycles per minute, or 0.1 hz) resulting in what is known as resonant frequency [55], resulting in a large multiplicative increase in the amplitude of HR oscillations and HRV power. Each individual has a resonant frequency [49] usually achieved at around 6 breaths per minute [51]. HRV BF involves training in abdominal breathing techniques, establishment of resonant frequency and monitoring over 10 weeks with home practice [55]. It has been linked to improvements in PTSD symptoms [18], [19], depression [56]–[59], state anxiety [56], [60], cardiovascular disease 17,61,62 and HRV levels [17], [19], [56], [60]. However, many of these studies used either no control group [57], [59], fail to control for experimenter contact [17], [60], [63], or introduce HRV BF along with emotion regulation strategies without controlling for the effects of these additional strategies [60], [63], highlighting the need for further research. When used in conjunction with cognitive treatments, HRV BF may improve emotion regulation capacity and thereby maximise the benefits of psychotherapy in treating anxiety [63].

A laboratory test designed to probe the nature of anxious response to performance must produce a period of anxious apprehension prior to performance, and potential for negative evaluation. Responses to this challenge can be measured using both HRV and self report measures. If biofeedback is effective in reducing anxiety, then vagal withdrawal and self reported anxiety should be reduced after receiving HRV BF. The Trier Social Stress Test (TSST) [64] is an established, standardised procedure recognised for producing physiological arousal in laboratory conditions [65] in response to the psychosocial stress of performing in front of an audience [66]. It involves a period of anxious apprehension prior to a speech on a difficult topic in front of a panel of judges. The anxious anticipation phase induces worry and preparation for future threat [67]. This technique has been used to explore elements of social phobia in clinical [68] and normal populations [69]. The TSST may be modified for musicians by replacing the speech component with a difficult musical performance task and requiring participants to look over the music and prepare their performance during an anticipation phase, a reliable stress response should be induced.

The aim of the current study was to explore the efficacy of HRV BF as an intervention for music performance anxiety. We hypothesised that, relative to control, the HRV BF and/or slow breathing intervention would: (1) reduce vagal withdrawal during anxious anticipation of a stressful performance task, leading to increased HRV during the anticipation phase following intervention; and (2) lead to reductions in self-reported state anxiety following musical performance. We demonstrate that a short slow breathing intervention increases HRV, and, amongst highly anxious participants, reduces anxiety.

## Methods

### Participants

46 musicians (24 females; aged 19–67, *M* = 30.4, *SD* = 11.98), trained as wind or brass players (n = 30), singers (n = 11), or string players (n = 5) (minimum grade 8 as determined by the Australian Music Examinations Board or tertiary level) participated in this study. All participants were required to abstain from food or drink for 2 hours prior to the experiment and caffeine on the day of experiment to control for the impact of these variables on HRV. Ethics approval was granted by the University of Sydney’s Human Research Ethics Committee (ref. 13702). All participants provided written informed consent in accordance with the Australian National Health and Medical Research Council guidelines.

### Materials

**Figure 1:**
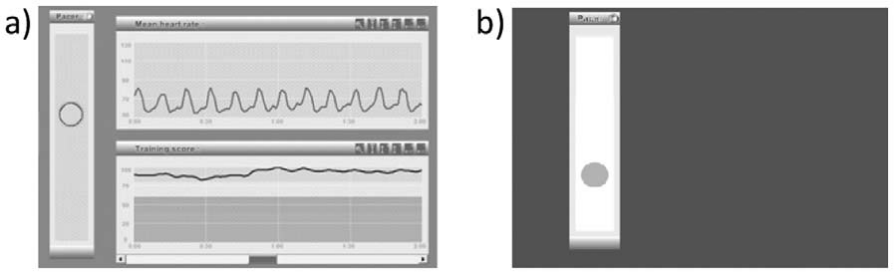
Screen shot of *Resilience Builder* software training screen and slow breathing pacer. a) The ball rises for inhalation and falls for exhalation with a longer interval for exhalation. The top graph shows HR, the large amplitude wave indicates slow deep breathing. The lower graph indicates score, which is very high in this case. b) Breathing pacer identical to Resilience Builder yet no feedback is provided.

HRV data was collected on the *Polar RS800CX* watch and chest-strap, which is a validated instrument for collecting HR and R-R interval data [70], [71], and event markers. HRV BF was administered on *Resilience Builder* HRV biofeedback software (*Innate Intelligence,* www.i-i.com.au/RB/index.html), run on an Acer Aspire 1 ZG5 laptop connected via USB to a finger pulse sensor (Biocom technologies pulse wave monitor HRM-02) a photoplethesmograph showing high (0.97) concordance with ECG measures [72]. The training component involved presenting a breathing pacer (Figure1); a small ball rising for inhalation and descending for exhalation at six breaths per minute, with extended expiration relative to inspiration. HRV feedback was provided via a colour-coded graph indicating low to high levels. HR feedback was provided in another graph above this.

Demographic information on smoking; past major health issues and mental illness; recent stimulant use (caffeine, amphetamines etc.); pregnancy; food or drink within last two hours and Body Mass Index (BMI) was collected. Information on recent physical activity (time spent doing vigorous and moderate physical exercise, sitting and walking in the last 7 days) was collected using the IPAQ [73]. The State-Trait Anxiety Inventory (revised STAI-Y; [74]) assessed anxiety. It is a brief, easily administered scale, consisting of 40 items coded on a 4-point Likert scale. It is a well validated, international instrument [75], sensitive to experimental manipulations of stress which discriminates between stable and transient factors contributing to anxiety [76]. The state subscale (STAI-S) measures transient emotional reactions. Scores range from 20 to 80, with a mean of approximately 34 and scores of 44 indicative of a highly anxious state [75], [77]. The STAI exhibits good internal consistency (α = 0.83–0.86); [78] and test-retest reliability (α = 0.73–0.86; Spielberger, 1983).

Musical tasks were created using Sibelius music notation software (Sibelius 6, version 6.0.1 build 5 audio engine vl.0145) and were designed to be very difficult for experienced sight readers. *Music task 1* involved sight singing a short, highly difficult, atonal musical passage; a modified excerpt of the orchestral flute part of *Le Marteau sans Maitre* [79]. The post intervention task was the same notes re-ordered. *Music task 2* involved sight reading, on instrument, a passage from one of two similar movements of *Quartet for the end of time* by Oliver Messiaen [80] to accompaniment performed on a Kawai grand piano, recorded on a Sony MD walkman MZ-NH700 mini-disc recorder and played back on computer through a Sony GRX10AV amplifier and DSE A2667 100W speakers. Performances were filmed on a Sony HDR-SR7E video recorder mounted on a Giottos VT-806 tripod. Participants were led to believe the recording would be used to assess performance quality. The true purpose of the camera, however, was to present the possibility of negative evaluation and to provide video for examination when anomalies (such as movement artefacts) in HRV recordings were observed.

### Procedure

The experiment was conducted in a sound attenuated room in the School of Psychology’s Psychophysiology Laboratory at the University of Sydney. Participants were assigned to group by a computer-generated list of random numbers, and after randomisation, fitted with the Polar HR monitor device. Testing was counterbalanced for time of day across group to control for known HRV circadian rhythms [81]. Recording began and experimental conditions were marked using time stamps on the Polar watch. All conditions were 5 minutes in accordance with HRV taskforce recommendations [30]. Screening questionnaires and baseline STAI-S were completed, then a five-minute resting state HRV recording was made while participants were sitting still, palms up, eyes open. *Anticipation phase*: Participants entered the testing room and were instructed to sit still and examine the music for five minutes in order to prepare for their performance. They were informed that their performance would be recorded on video camera while the experimenter observed. Participants were told to prepare for the performance ahead, concentrating on accuracy and musical interpretation, to the best of their ability. The experimenter then left the room. *Performance phase:* Participants were instructed to begin the performance without stopping in the event of a mistake and to remember that interpretive quality was important. Following the testing phase participants moved to the intervention room and completed STAI-S questionnaires. Diaphragmatic breathing was demonstrated and participants completed a number of breathing exercises. *Intervention phase:* Intervention lasted 30 minutes. The biofeedback and breathing control group were given diaphragmatic breating instruction. The biofeedback group received a modified version of a biofeedback procedure previously validated by Lehrer and colleagues [55]. Participants were instructed to increase RSA by breathing with the pacer in order to increase the amplitude of HR fluctuations to achieve the best possible feedback graph score while breathing in a free, relaxed way. The breathing control group were given all the same breathing instructions and breathed to a functionally identical pacer without feedback. The no-intervention control group were simply instructed to read their preferred reading material. Post-intervention testing featured exactly the same procedure as the pre-intervention anticipation and performance phases with counterbalanced novel musical stimuli followed by STAI-S completion.

### Data and Statistics

Text files of the R-R interval data were extracted from the Polar watch and were entered into Kubios HRV (version 2.0, 2008, biosignal analysis and medical imaging group, University of Kuopio, Finland, MATLAB). Five-minute resting baseline and 5-minute anticipation phases were then filtered with the low automatic filter and visually inspected for artefact. Interbeat intervals (IBI) varying more than 20% from the previous interval [30] were replaced by linear interpolation. Two participants with large amounts of artefact were excluded. Upon examination of HRV variables, an additional participant was identified as an outlier in multiple univariate analyses with z scores exceeding the conventional cut-off of 3.29 [82] and was excluded from the analysis.

### Statistical Analysis

Statistical tests were conducted using IBM SPSS Statistics 19. A one way ANOVA was conducted to detect any between group differences in resting state HRV. 3 group (HRV BF, Slow Breathing, No Treatment Control)×(2) time (pre, post intervention), mixed design ANCOVAs were then conducted on both HF and LF/HF ratio measures of HRV. Time spent walking in the past seven days was added as a covariate in order to control for the effect of recent exercise on HRV [83]. A 3 group (HRV BF, Slow Breathing, No Treatment Control) ×(2) time (pre, post intervention), mixed design ANCOVA was also conducted with STAI-S scores as the dependent variable. Baseline STAI-S scores were entered as a covariate in order to control for individual differences in state anxiety. The between-subject factor was whether participants received slow, paced breathing plus biofeedback; slow, paced breathing alone or no-treatment control (book reading). Planned contrasts were conducted on all mixed design ANCOVAs to examine differences between the two intervention groups as well as between the intervention and control. The two within-subjects levels were pre- and post-intervention scores. Statistical significance level was set at 0.05 (one-tailed; pairwise comparisons with directional hypotheses).

## Results

### Participant Characteristics

**Figure 2:**
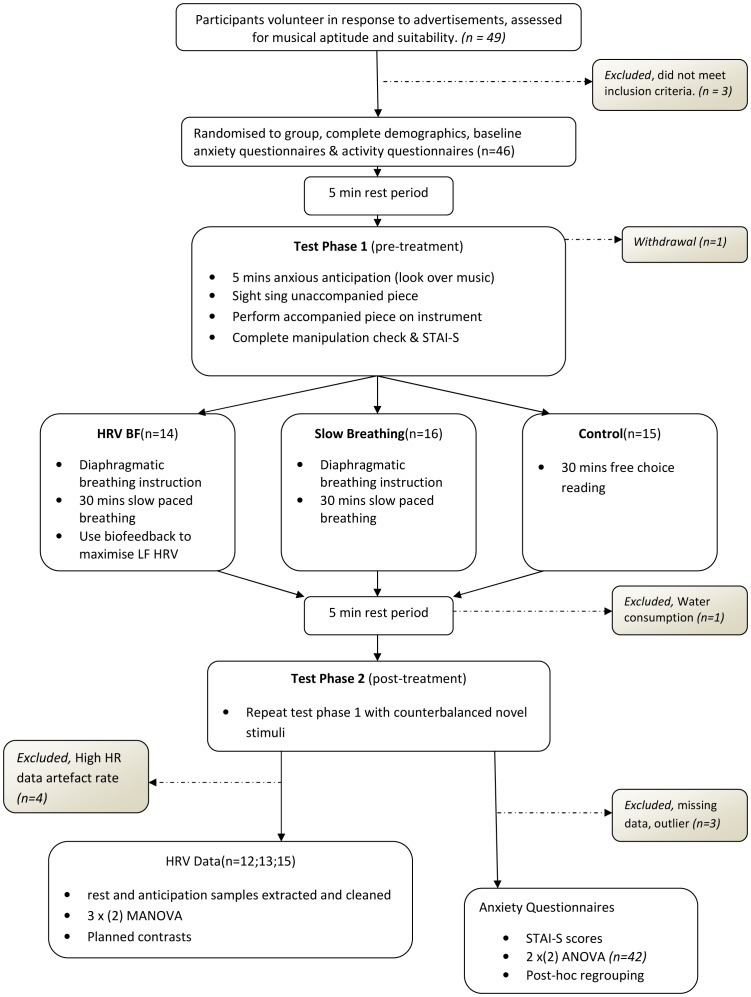
Flow diagram of participant progress through the study. Figure depicts progress through the stages of the experiment, including when and why participants were excluded. Conforms to CONSORT guidelines [89].

Demographics are presented in Table 1. See Figure 2 for progress of participants through the study. While the intervention groups displayed higher STAI-T scores than controls, (*F*(2,41) = 4.497, *p* = 0.017), and a greater frequency of reporting history of mental illness (*F*(2,38) = 3.694, p = 0.034), no differences were observed on baseline resting state HRV(HF: *F* = 0.294, *p* = 0.591, LF/HF: *F* = 0.231, *p* = 0.633) or baseline state anxiety (*F*(1,42) = 1.316, *p* = 0.258). Graphs were visually inspected to determine whether mental illness history had a systematic effect on dependent variables. Individuals with a history of mental illness were evenly spread on all measures except LF/HF ratio on which one participant, with a history of mental illness, scored considerably higher. Since these scores did not fulfil the criteria for outliers outlined above, and the participant appeared normal on the other four measures, they were not excluded from the analysis. Higher levels of both trait anxiety (Kenny, 2004), self-reported mental health history (Marchant-Haycox, 1992) and higher reporting of psychological symptoms (Zander, 2010) have been reported in musicians as compared to normative samples. Consequently, participants with a history of mental illness were not removed from analyses to maintain a representative sample of musicians. Instead, further analyses were conducted to understand the role of mental illness in the findings obtained (described below).

**Table 1:** Participant characteristics.

			Group								
			HRV BF (n = 14)		Breathing (n = 15)		Control (n = 15)				
Participant Charactersitics			Mean	*SD*	Mean	*SD*	Mean	*SD*	*df*	*F*	sig.
Age			29.42	7.56	31.86	2.7	32.93	14.81	2,38	0.27	0.77
Body mass Index			24.26	4.56	23.58	3.69	21.55	1.74	2,36	2.31	0.11
PANAS negative			12.07	4.10	15.00	5.99	12.20	2.93	2, 41	1.976	0.15
PANAS positive			29.21	10.928	33.73	3.058	29.93	6.319	2, 41	1.589	0.216
STAI-S			34.64	8.308	32.67	7.835	30.4	10.308	2, 41	0.827	0.445
STAI-T			45.07	8.722	42.13	9.07	35.53	8.626	2, 41	4.497	0.017
Exercise in the last 7 days											
Vigorous (mins)			122.73	105.39	51.43	58.16	99.67	107.31	2,37	1.99	0.15
Moderate (mins)			56.91	75.67	61.5	65.64	80.87	78.69	2,37	0.409	0.67
Hours sitting			6.82	2.35	5.57	3.43	6.39	2.98	2,36	0.57	0.57
Hours walking			2.8	2.38	4.54	2.63	4.5	2.86	2,37	1.69	0.2
			Frequency		Frequency		Frequency		*df*	***X^2^***	sig.
Gender	Males		6		7		7				
	Females		6		7		8		2	0.04	0.98
History of Mental Illness			4		5		0		2	5.953	0.05^a^
Past Medical Problems			4		4		2		2	2.25	0.33^a^
Caffeine in last 24 hrs			4		4		6		2	0.59	0.74^a^
Using medication			3		2		2		2	1.59	0.56^a^
Smoker			2		1		2		2	8.68	0.65^a^

### Validation of Anxiety-Provoking Task and the Intervention

**Figure 3:**
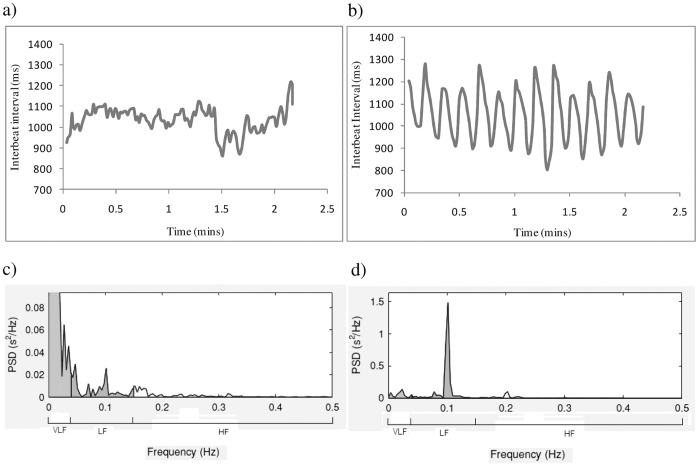
Individual Data: Interbeat intervals (IBI) and frequency analysis during Rest period and Slow breathing. a) IBI during two minutes of resting state. Changes in HR due to respiration, 12–14 breaths per minute, are small and irregular. b) IBI during two minutes of slow breathing. Changes in HR due to respiration, 6 breathes per minute, are large and regular. High amplitude indicates large differences between the slowest beats during exhalation and fastest beats during inhalation. c) Frequency analysis during 5 minutes resting state indicates low amplitude LF and HF (recording is too short to accurately characterise VLF (Malick, 1996)) d) Frequency analysis during 5 minutes slow breathing indicates very high amplitude LF oscillations at the frequency of breathing (10 times per minute or 0.1 Hz).

Overall, participants exhibited anxiety symptoms in response to the anxiety-provoking performance task, showing significant reductions in HF from resting state to anxious anticipation, *F*(1,34) = 4.165, *p* = 0.049, and significant increases in STAI – S scores, *F*(1,39) = 29.069, *p*<0.001 regardless of intervention. Examination of individual data revealed large increases in RSA amplitude during the slow breathing tasks (Figure 3), consistent with the expected effects of the intervention.

### HRV during Anxious Anticipation

**Figure 4:**
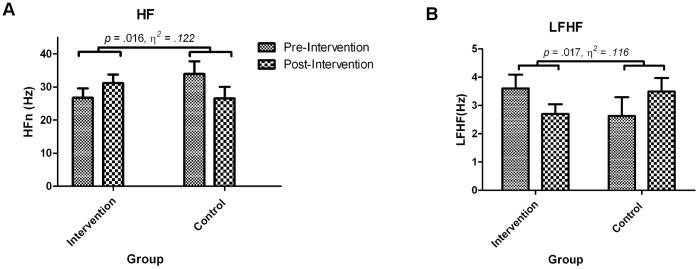
Group differences in HRV comparing intervention to control. Group means and standard error for HRV measures during anxious anticipation pre and post intervention. Both HF and LF/HF ratio showed significant group by time interactions. a) Participants in the intervention group increased HF after intervention, whereas those in the control group decreased. b) Participant in the intervention group decreased LF/HF ratio whereas those in the control group increased.

3 Group (HRV BF, Slow Breathing, No Treatment Control)×(2) Time (pre, post intervention) mixed design ANCOVAs were employed for HF and LF/HF ratio with time spent walking as a covariate. There were no significant main effects for time, group or interaction between group and time. Planned contrasts revealed a significant interaction when comparing intervention groups (HRV BF and breathing) to control, HF: *t*(38) = 2.32, *p* = 0.026 (see Table 2), LF/HF: *t*(38) = 2.21, *p* = 0.017.These findings (Figure 4 and Table 2) indicate an increase in vagal tone in the intervention groups (HRV BF and slow breathing) but a decrease in vagal tone in the control group in the post intervention relative to pre intervention assessment.

**Table 2:** High Frequency (Natural log) Means and Standard Error.

			Group					
			HRV BF (n = 14)		Breathing (n = 15)		Control (n = 15)	
HF (log)			Mean	*SE*	Mean	*SE*	Mean	*SE*
Pre-intervention Anticipation			3.219	.146	3.219	.131	3.428	.122
Post-intervention Anticipation			3.419	.127	3.298	.114	3.205	.106

### Behavioural Data: State Anxiety

Two participants with missing data were excluded. A 3 Group (HRV BF, slow breathing, control)×(2) time (pre-, post-intervention) mixed design ANCOVA was conducted with pre and post intervention STAI-S scores as the dependent variables and baseline STAI-S as a covariate to control for individual differences. There were no significant main effects for time, group or interaction between group and time. Planned contrasts revealed no significant interaction when comparing intervention groups (HRV BF and breathing) to control, intervention *MD* = 5.31; control *MD* = 1.082; *t*(38) = 1.56, *p* = 0.064, or when comparing HRV BF to breathing groups, *t*(38) = 0.38, *p* = 0.348. Those who received intervention showed significantly decreased STAI-S scores following intervention, *t*(df) = 3.33, *p* = 0.001 where as those in the control group showed negligible differences, *t(df)* = 0.5, *p* = 0.314.

### Post-Hoc Analyses

#### Interaction between Group and Pre-intervention Anxiety

We conducted additional post hoc regression analyses to determine whether pre-existing state anxiety levels in response to performance impacted on change in state-anxiety following intervention. It was hypothesised that high-anxious individuals in the breathing group would show large reductions in STAI-S scores from pre to post intervention whereas low-anxious individuals would show a floor effect, i.e., already low anxiety scores would remain low. Pre-intervention STAI-S scores were entered as the first step in a hierarchical regression - this is a more accurate measure of music performance anxiety than baseline STAI-S since it is in response to music performance, predicting STAI-S pre-post difference scores. Their interaction term was then added. Pre-intervention STAI-S scores significantly moderated the relationship between group and pre-post anxiety difference scores (*β* = 0.46, *F change* (1,38) = 4.92, *p = *0.033) confirming our hypothesis.

Simple slopes revealed a significant linear relationship between pre-intervention STAI-S and STAI-S change scores in the intervention group (*β* = 0.59, *F*(1,25) = 12.99, *p = *0.001), but not in the control group (*β* = 0.11, *F*(1,13) = 0.17, *p = *0.69). These findings indicate that individuals exhibiting higher anxiety initially tended to report greater reductions in anxiety after the intervention phase and this relationship was significantly different than that in the control group.

#### Between group differences amongst high-anxious individuals

The interaction between existing anxiety and intervention indicated a floor effect amongst low anxious individuals, which may have masked a clinically-relevant intervention effect in high anxious individuals. In order to compare groups with similar levels of pre-existing anxiety, groups were subdivided into high and low anxiety by median split. Comparison of observed median to population norms for the STAI-S (*M = *34.2) [77], [84] indicated that the median of 43 represents a clinically meaningful cut-off point. The high-anxious participants in intervention and control groups had similar (high) levels of pre-intervention state anxiety (intervention group: *M = *53.1; control *M = *52.3). It was hypothesised that, amongst high-anxious individuals, the intervention would result in significantly greater pre-post reductions in STAI-S scores.

**Figure 5:**
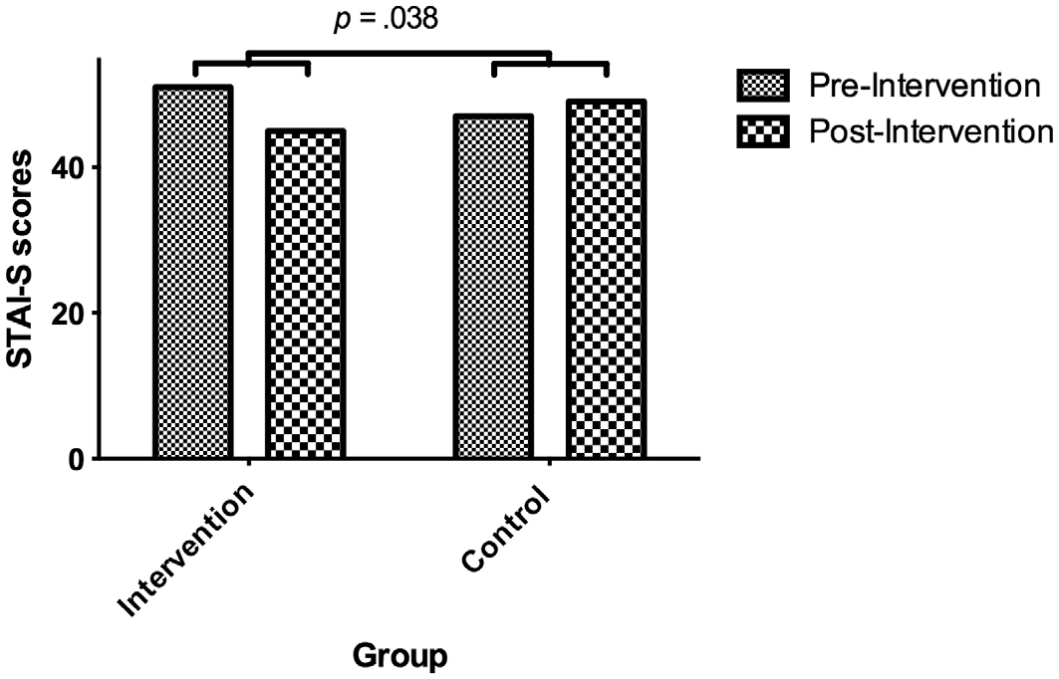
Group medians of STAI-S scores before and after intervention amongst highly anxious individuals. Amongst highly anxious individuals, participants in the intervention group showed a reduction in reported state anxiety while those in the control group showed a modest increase. The difference between pre-post difference scores for each group was significant (i.e. a group by time interaction).

The non-parametric Mann-Whitney U was used, due to small sample size, to test the difference in STAI-S reductions (change scores) between the intervention and control groups amongst high anxious individuals. Participants in the intervention group (*Mdn* = 7) exhibited a significantly greater reduction in STAI-S scores than those in the control group (*Mdn* = −1), *U* = 21.5, *p* = 0.045, *r = *0.379 (one-tailed) (Figure 5).

**Figure 6:**
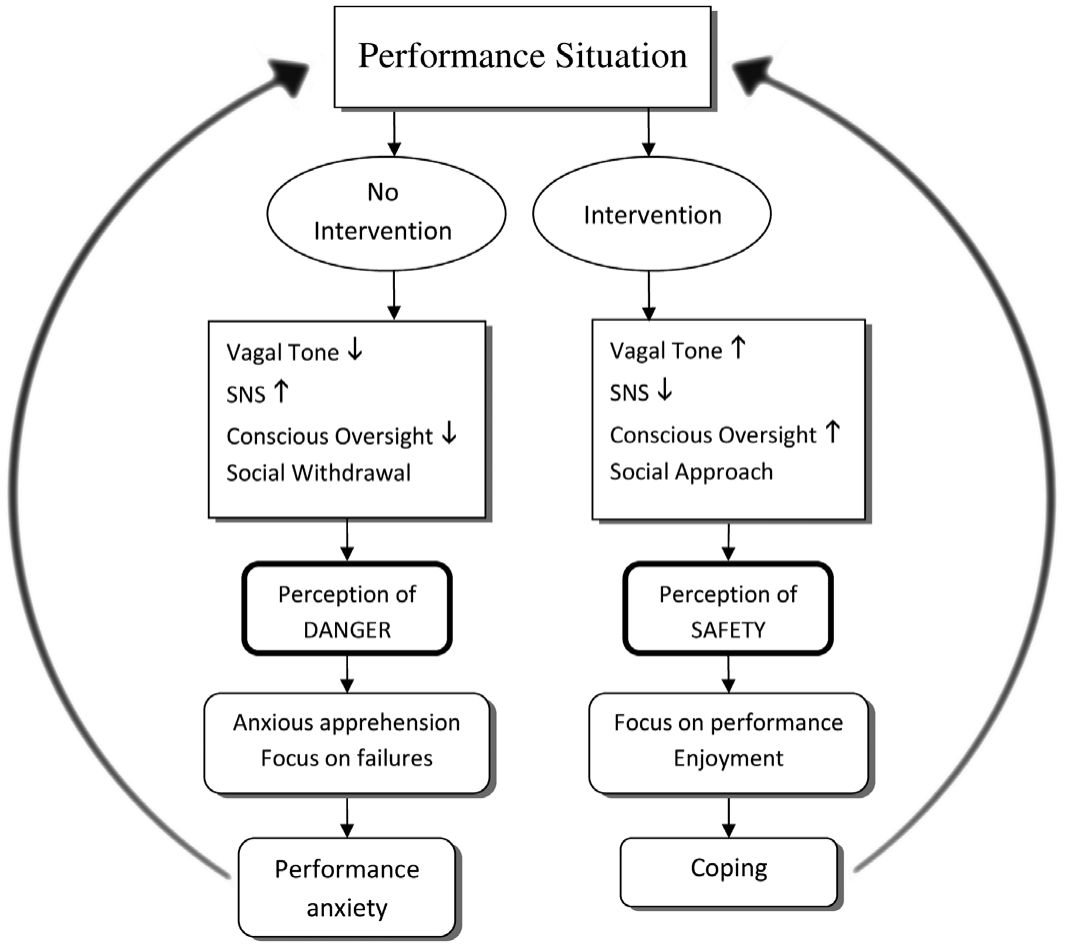
The role of cognitive and physiological factors in the development of MPA. The interaction of cognitive and emotional factors in the etiology of music performance anxiety. The performance situation is interpreted in line with previous performance experience and cognitive factors, such as pressure to perform. Slow breathing intervention influences level of vagal tone, inhibiting immediate arousal and enhancing approach tendencies. These physiological factors feedback to cortical areas to influence further interpretation of the environment as either threatening or safe. Perceived safety determines behaviour and interpretation of performance, which leads to either dysregulation and anxiety or successful regulation and coping behaviours. Level of anxious response determines future interpretations of performance environment.

## Discussion

The results of this study provide evidence for the efficacy of a slow breathing protocol: HRV levels were increased in response to stress, and state anxiety reduced in anxious individuals. The aim of the study was to examine whether a single session of slow breathing (with or without HRV BF) was effective in reducing both vagal withdrawal and state anxiety in response to the psychosocial stress of musical performance. Participants who received slow breathing exhibited greater increases in HF and decreases in LF/HF ratio than controls after intervention, indicating increased levels of parasympathetic influence on HR while under stress. This increased level of PNS inhibition may allow participants to better regulate physiological arousal prior to music performance and to perform more competently. Amongst highly anxious individuals, the intervention also led to greater reductions in self-reported anxiety than controls, indicating that slow breathing may have clinically-relevant effects for performing musicians who suffer from anxiety.

The anticipation phase of this experiment mimicked the conditions that can lead to MPA. The presence of the experimenter created the possibility for negative evaluation while the high difficulty of the tasks made it likely that participants would make mistakes, inducing physiological and behavioural symptoms of performance anxiety, indicated by marked HF reductions and self-reported increases in state anxiety respectively. Under these stressful conditions, intervention produced increases in HRV as well as decreases in self-reported anxiety in high anxious individuals.

Slow breathing, with or without BF, produced differential effects on both HF and LF/HF after intervention compared to controls. Any potential differences produced by the addition of biofeedback were not apparent after a single session. Those who received the slow breathing intervention (relative to controls) showed marked increases in HF, indicating increases in vagal influence on HR during anticipation of a socially stressful and technically challenging musical performance. These increases were observed despite the effects of mental fatigue, which is known to cause decreases in HF [85]. This is also a likely explanation for HF decreases in the control group. While the control group showed increases in LF/HF ratio, indicating increased LF relative to HF levels, the intervention group showed decreased levels of LF relative to HF power during the anticipation phase following intervention.

LF/HF ratio indicates the amount of HRV that is due to a combination of SNS, PNS and blood pressure mechanisms (LF) relative to vagal input (HF) under normal conditions. It should be noted that LF levels are also inflated by slow breathing, especially when breathing at a rate of 0.1 Hz, which is in the LF range. Breathing at this rate creates a resonant effect whereby oscillations of vagal outflow are timed to coincide with those in baroreflex action, producing large increases in the amplitude of HR changes [55]. Our LF/HF ratio findings under anticipation stress indicate that participants receiving the intervention were not simply continuing to breathe at 0.1 Hz (LF). Rather, the increases in HRV amplitude at the rate of normal breathing (HF) were greater. The HRV amplitude increases associated with the intervention were maintained under normal breathing conditions, and in the face of psychological challenge. Current thinking places LF activity as activity of the baroreflex [86], [87] and therefore calls into question the direct relationship between LF/HF ratio and SNS/PNS balance (eg. [88]); while our findings do not provide evidence on this subject, our findings do indicate that reductions in LF/HF during stress are associated with state-anxiety reductions.

We also observed a relationship between the level of state anxiety prior to the intervention and state anxiety after the intervention. Regression analyses revealed that initially high anxious individuals tended to show reductions in state anxiety after intervention. By contrast, no effects were observed in those with low state anxiety before the intervention. Regrouping removed this confounding factor and allowed intervention and control participants of similar levels of anxiety to be compared. This finding highlights the potential for slow breathing to reduce state anxiety in musicians who experience anxiety in performance situations.

These findings support a model of MPA in which autonomic regulation impacts on the experience of anxiety in response to music performance while highlighting the effectiveness of slow breathing as an intervention which can lead to clinically relevant reductions in anxiety. Higher levels of vagal tone are associated with greater executive control and ability to inhibit fear-related emotional responses through cortical inhibition of lower limbic structures [35]; and enable social engagement through bi-directional feedback between heart and brain [21]. Individuals with a high level of vagal tone display greater flexibility in response to challenges and are able to effectively inhibit SNS activity associated with stress [38]. This increased autonomic flexibility allows for the effective regulation of negative emotion in the face of stress [22] which is, in turn, required for the amelioration of anxiety. MPA may arise from acute awareness of the possibility for negative evaluation leading to physiological symptoms which subsequently interfere with successful performance [3]. Impacting on physiological feedback mechanisms through slow breathing helps to inhibit threat-related arousal, reduce threat appraisal and break the MPA cycle (Figure 6).

A variety of questions regarding the role of breathing rate in performance anxiety are raised by this study, for which measurement of breathing rate may help to clarify in future studies. It may be the case that those who reported high anxiety in response to initial performance showed dysregulated breathing as found by Studer et al. [11]. It may be that hyperventilation in anticipation of performance potentiates physiological anxiety and that either the abdominal breathing training or the slow paced breathing prevented this from occurring after intervention. Breathing rate during anticipation could be compared with HRV and state anxiety findings to determine if better regulated breathing (ie. slower, deeper breathes) leads to both HRV increases and state anxiety decreases. Even if the observed effect of slow breathing is purely due to placebo effect, since it was evident to participants that they were receiving an intervention, the intervention comes with so few costs, that its use may be justified. There are no known side effects, it requires no expensive equipment or special knowledge, it has no financial cost, and it is non-pharmacological. Pharmacological treatments, such as beta-blockers can have adverse mood effects [14]. Slow breathing also has the added benefits of increased oxygen absorption and lowered blood pressure [48], [50]. The findings of this preliminary trial warrant further longitudinal investigation in order to better determine the effect of slow breathing on MPA, particularly in samples of those with clinical levels of MPA.

This study did not include important components of a full HRV BF treatment, such as establishment of resonant frequency and a 10 week course. There may be additional effects which only become apparent with practice. Previous research has shown that longer courses of HRV BF can result in significantly greater HRV increases relative to relaxation techniques [19]. If there is a therapeutic effect of HRV BF above relaxation produced by slow breathing then this may only become apparent after a full course of treatment. This possibility remains to be tested. An important component omitted in the present study was the establishment of individual resonant frequency. Lehrer et al. [55] argue that resonant frequency calculation is integral to the therapeutic effect of HRV BF since maximal HRV is produced at resonant frequency. Previous studies, which have calculated resonant frequency prior to commencing treatment, have reported results supporting the efficacy of HRV BF [17], [18], [58], [60]. The establishment of resonant frequency is a time consuming process, which was not practical in the present, single session experiment. It is possible that differences between HRV BF and slow breathing may emerge when resonant frequency is calculated for individual participants.

In summary, the results of the present study indicate that diaphragmatic breathing instruction and a single session of slow breathing are sufficient to produce HRV increases and state anxiety reductions among musicians with high state anxiety, and that biofeedback is not necessary for these changes to occur. This suggests that integration of slow breathing with more comprehensive psychotherapy strategies may have clinical utility in the treatment of individuals with MPA. Slow breathing may be a viable alternative to beta-blockers for inhibition of SNS activity during performance. Future studies could compare slow breathing to beta-blockers and placebo treatments such as paced breathing at a normal rate (approximately 15 breathes per minute [48]). A longitudinal study may help determine whether the addition of biofeedback leads to better results with practice.

## References

[1] Ostwald PF, Baron BC, Byl NM, Wilson FR (1994) Performing arts medicine. West J Med 160: 48–52. 8128702 PMC1022254

[2] Robson BE Gillies E (1987) Post-performance depression in arts students. Med Probl Performing Art: 137–141.

[3] Kenny D (2011) The Psychology of Music Performance Anxiety. New York: Oxford University Press. 376 p.

[4] Bodner E (2008) After the Curtain Falls: On the Post-Performance Adjustment of Solo Singers. Medical Problems of Performing Artists 23: 172–177.

[5] Zander MF, Voltmer E, Spahn C (2010) Health promotion and prevention in higher music education: results of a longitudinal study. Med Probl Perform Art 25: 54–65. 20795333

[6] Wesner RB, Noyes Jr R, Davis TL (1990) The occurence of performance anxiety among musicians. Journal of Affective Disorders 18: 177–185. 2139062 10.1016/0165-0327(90)90034-6

[7] Steptoe A, Fidler H (1987) Stage fright in orchestral musicians: A study of cognitive and behavioural strategies in performance anxiety. British Journal of Psychology 78: 241–249. 3594093 10.1111/j.2044-8295.1987.tb02243.x

[8] van Kemenade JF, van Son MJ, van Heesch NC (1995) Performance anxiety among professional musicians in symphonic orchestras: a self-report study. Psychological reports 77: 555–562. 8559881 10.2466/pr0.1995.77.2.555

[9] Kenny DT, Davis P, Oates J (2004) Music performance anxiety and occupational stress amongst opera chorus artists and their relationship with state and trait anxiety and perfectionism. Journal of Anxiety Disorders 18: 757–777. 15474851 10.1016/j.janxdis.2003.09.004

[10] Brantigan T, Brantigan C, Joseph N (1978) Beta-blockade and musical performance. The Lancet 312: 896. 10.1016/s0140-6736(78)91605-7 81441

[11] Studer R, Danuser B, Hildebrandt H, Arial M, Gomez P (2011) Hyperventilation complaints in music performance anxiety among classical music students. Journal of Psychosomatic Research 70: 557–564. 21624579 10.1016/j.jpsychores.2010.11.004

[12] Steptoe A (1989) Stress, Coping and Stage Fright in Professional Musicians. Psychology of Music 17: 3–11.

[13] Hayes PE, Schulz SC (1987) Beta-blockers in anxiety disorders. Journal of Affective Disorders 13: 119–130. 2890677 10.1016/0165-0327(87)90017-6

[14] Head A, Kendall MJ, Ferner R, Eagles C (1996) Acute effects of beta blockade and exercise on mood and anxiety. Br J Sports Med 30: 238–242. 8889119 10.1136/bjsm.30.3.238 PMC1332339

[15] Fehm L, Schmidt K (2006) Performance anxiety in gifted adolescent musicians. Journal of Anxiety Disorders 20: 98–109. 16325117 10.1016/j.janxdis.2004.11.011

[16] Dobson MC (2011) Insecurity, professional sociability, and alcohol: Young freelance musicians perspectives on work and life in the music profession. Psychology of Music 39: 240–260.

[17] Del Pozo JM, Gevirtz RN, Scher B, Guarneri E (2004) Biofeedback treatment increases heart rate variability in patients with known coronary artery disease. American Heart Journal 147: 545. 10.1016/j.ahj.2003.08.013 14999213

[18] Tan G, Dao T, Farmer L, Sutherland R, Gevirtz R (2011) Heart Rate Variability (HRV) and Posttraumatic Stress Disorder (PTSD): A Pilot Study. Applied Psychophysiology and Biofeedback 36: 27–35. 20680439 10.1007/s10484-010-9141-y

[19] Zucker T, Samuelson K, Muench F, Greenberg M, Gevirtz R (2009) The Effects of Respiratory Sinus Arrhythmia Biofeedback on Heart Rate Variability and Posttraumatic Stress Disorder Symptoms: A Pilot Study. Applied Psychophysiology and Biofeedback 34: 135–143. 19396540 10.1007/s10484-009-9085-2

[20] Prinsloo GE, Rauch HGL, Lambert MI, Muench F, Noakes TD, et al (2011) The Effect of Short Duration Heart Rate Variability (HRV) Biofeedback on Cognitive Performance During Laboratory Induced Cognitive Stress. Applied Cognitive Psychology 25: 792–801.

[21] Porges SW (2011) The polyvagal theory : neurophysiological foundations of emotions, attachment, communication, and self-regulation. New York: Norton. 347 p.

[22] Appelhans BM, Luecken LJ (2006) Heart rate variability as an index of regulated emotional responding. Review of General Psychology 10: 229–240.

[23] Zulfiqar U, Jurivich DA, Gao W, Singer DH (2010) Relation of High Heart Rate Variability to Healthy Longevity. The American Journal of Cardiology 105: 1181–1185. 20381674 10.1016/j.amjcard.2009.12.022

[24] Kemp AH, Quintana DS, Gray MA, Felmingham KL, Brown K, et al (2010) Impact of Depression and Antidepressant Treatment on Heart Rate Variability: A Review and Meta-Analysis. Biological Psychiatry 67: 1067–1074. 20138254 10.1016/j.biopsych.2009.12.012

[25] Thayer JF, Friedman BH, Borkovec TD (1996) Autonomic characteristics of generalized anxiety disorder and worry. Biological Psychiatry 39: 255–266. 8645772 10.1016/0006-3223(95)00136-0

[26] Gorman JM, Sloan RP (2000) Heart rate variability in depressive and anxiety disorders. American Heart Journal 140: 77–83. 11011352 10.1067/mhj.2000.109981

[27] Kemp AH, Quintana DS, Felmingham KL, Matthews S, Jelinek HF (2012) Depression, Comorbid Anxiety Disorders, and Heart Rate Variability in Physically Healthy, Unmedicated Patients: Implications for Cardiovascular Risk. PLoS ONE 7: e30777. 22355326 10.1371/journal.pone.0030777 PMC3280258

[28] Karemaker JM, Lie KI (2000) Heart rate variability: a telltale of health or disease. European Heart Journal 21: 435–437. 10681483 10.1053/euhj.1999.1969

[29] Geisler FCM, Vennewald N, Kubiak T, Weber H (2010) The impact of heart rate variability on subjective well-being is mediated by emotion regulation. Personality and Individual Differences 49: 723–728.

[30] Malik M, Bigger JT, Camm AJ, Kleiger RE, Malliani A, et al (1996) Heart rate variability. European Heart Journal 17: 354–381.8737210

[31] Pumprla J, Howorka K, Groves D, Chester M, Nolan J (2002) Functional assessment of heart rate variability: physiological basis and practical applications. International Journal of Cardiology 84: 1–14. 12104056 10.1016/s0167-5273(02)00057-8

[32] Berntson GG, Thomas Bigger J, Eckberg DL, Grossman P, Kaufmann PG, et al (1997) Heart rate variability: Origins, methods, and interpretive caveats. Psychophysiology 34: 623–648. 9401419 10.1111/j.1469-8986.1997.tb02140.x

[33] Grossman P, Taylor EW (2007) Toward understanding respiratory sinus arrhythmia: Relations to cardiac vagal tone, evolution and biobehavioral functions. Biological Psychology 74: 263–285. 17081672 10.1016/j.biopsycho.2005.11.014

[34] Lopes P, White J (2006) Heart rate variability: measurement and practical implications. In: Peter J. Maud CF, editor. Physiological assessment of human fitness 2nd ed.

[35] Lane RD, McRae K, Reiman EM, Chen K, Ahern GL, et al (2009) Neural correlates of heart rate variability during emotion. NeuroImage 44: 213–222. 18778779 10.1016/j.neuroimage.2008.07.056

[36] Malliani A (2005) Heart rate variability: from bench to bedside. European Journal of Internal Medicine 16: 12–20. 15733815 10.1016/j.ejim.2004.06.016

[37] Gevirtz R (2007) Psychophysiological Perspectives on Stress-Related and Anxiety Disorders. In: Paul Lehrer RWWS, editor. Principles and Practice of Stress Management. 3rd ed. New York: The Guildford Press. 209–226.

[38] Friedman BH (2007) An autonomic flexibility-neurovisceral integration model of anxiety and cardiac vagal tone. Biological Psychology 74: 185–199. 17069959 10.1016/j.biopsycho.2005.08.009

[39] Thayer JF, Hansen AL, Saus-Rose E, Johnsen BH (2009) Heart Rate Variability, Prefrontal Neural Function, and Cognitive Performance: The Neurovisceral Integration Perspective on Self-regulation, Adaptation, and Health. Annals of Behavioral Medicine 37: 141–153. 19424767 10.1007/s12160-009-9101-z

[40] Thayer JF, Lane RD (2000) A model of neurovisceral integration in emotion regulation and dysregulation. Journal of Affective Disorders 61: 201–216. 11163422 10.1016/s0165-0327(00)00338-4

[41] Thayer JF, Lane RD (2009) Claude Bernard and the heart-brain connection: Further elaboration of a model of neurovisceral integration. Neuroscience & Biobehavioral Reviews 33: 81–88. 18771686 10.1016/j.neubiorev.2008.08.004

[42] Porges SW (2003) Social Engagement and Attachment. Annals of the New York Academy of Sciences 1008: 31–47. 14998870 10.1196/annals.1301.004

[43] Campbell-Sills L, Barlow DH (2007) Incorporating Emotion Regulation into Conceptualizations and Treatments of Anxiety and Mood Disorders. In: Gross JJ, editor. Handbook of emotion regulation: Guildford Press. 676.

[44] Gross JJ, Thompson RA (2007) Emotion Regulation, Conceptual Foundations. In: Gross JJ, editor. Handbook of emotion regulation: The Guildford Press. 673.

[45] Thompson RA, Lewis MD, Calkins SD (2008) Reassessing Emotion Regulation. Child Development Perspectives 2: 124–131.

[46] Mennin D (2006) Emotion Regulation Therapy: An Integrative Approach to Treatment-Resistant Anxiety Disorders. Journal of Contemporary Psychotherapy 36: 95–105.

[47] Wilhelm FH, Gevirtz R, Roth WT (2001) Respiratory Dysregulation in Anxiety, Functional Cardiac, and Pain Disorders. Behavior Modification 25: 513–545. 11530714 10.1177/0145445501254003

[48] Joseph C, Porta C, Casucci G, Casiraghi N, Maffeis M, et al (2005) Slow Breathing Improves Arterial Baroreflex Sensitivity and Decreases Blood Pressure in Essential Hypertension. Hypertension 46: 714–718. 16129818 10.1161/01.HYP.0000179581.68566.7d

[49] Lehrer P (2003) Applied Psychophysiology: Beyond the Boundaries of Biofeedback (Mending a Wall, a Brief History of Our Field, and Applications to Control of the Muscles and Cardiorespiratory Systems). Applied Psychophysiology and Biofeedback 28: 291–304. 14686082 10.1023/a:1027330909265

[50] Bernardi L, Porta C, Spicuzza L, Bellwon J, Spadacini G, et al (2002) Slow Breathing Increases Arterial Baroreflex Sensitivity in Patients With Chronic Heart Failure. Circulation: Journal of the American Heart Association 105: 143–145. 10.1161/hc0202.103311 11790690

[51] Hirsch JA, Bishop B (1981) Respiratory sinus arrhythmia in humans - How breathing pattern modulates heart-rate. American Journal of Physiology 241: H620–H629. 7315987 10.1152/ajpheart.1981.241.4.H620

[52] Bernardi L, Passino C, Wilmerding V, Dallam G, Parker D, et al (2001) Breathing patterns and cardiovascular autonomic modulation during hypoxia induced by simulated altitude. Journal of Hypertension 19: 947–958. 11393679 10.1097/00004872-200105000-00016

[53] Bernardi L, Sleight P, Bandinelli G, Cencetti S, Fattorini L, et al (2001) Effect of rosary prayer and yoga mantras on autonomic cardiovascular rhythms: comparative study. BMJ 323: 1446–1449. 11751348 10.1136/bmj.323.7327.1446 PMC61046

[54] Tzeng YC, Sin PYW, Lucas SJE, Ainslie PN (2009) Respiratory modulation of cardiovagal baroreflex sensitivity. Journal of Applied Physiology 107: 718–724. 19608928 10.1152/japplphysiol.00548.2009

[55] Lehrer PM, Vaschillo E, Vaschillo B (2000) Resonant Frequency Biofeedback Training to Increase Cardiac Variability: Rationale and Manual for Training. Applied Psychophysiology and Biofeedback 25: 177–191. 10999236 10.1023/a:1009554825745

[56] Siepmann M, Aykac V, Unterdörfer J, Petrowski K, Mueck-Weymann M (2008) A Pilot Study on the Effects of Heart Rate Variability Biofeedback in Patients with Depression and in Healthy Subjects. Applied Psychophysiology and Biofeedback 33: 195–201. 18807175 10.1007/s10484-008-9064-z

[57] Karavidas M, Lehrer P, Vaschillo E, Vaschillo B, Marin H, et al (2007) Preliminary Results of an Open Label Study of Heart Rate Variability Biofeedback for the Treatment of Major Depression. Applied Psychophysiology and Biofeedback 32: 19–30. 17333315 10.1007/s10484-006-9029-z

[58] Nolan RP, Kamath MV, Floras JS, Stanley J, Pang C, et al (2005) Heart rate variability biofeedback as a behavioral neurocardiac intervention to enhance vagal heart rate control. American Heart Journal 149: 1137. 10.1016/j.ahj.2005.03.015 15976804

[59] Hassett AL, Radvanski DC, Vaschillo EG, Vaschillo B, Sigal LH, et al (2007) A Pilot Study of the Efficacy of Heart Rate Variability (HRV) Biofeedback in Patients with Fibromyalgia. Applied Psychophysiology and Biofeedback 32: 1–10. 17219062 10.1007/s10484-006-9028-0

[60] Thurber MR (2006) Effects of heart-rate variability biofeedback training and emotional regulation on music performance anxiety in university students [Ph.D.]. United States – Texas: University of North Texas.

[61] Swanson K, Gevirtz R, Brown M, Spira J, Guarneri E, et al (2009) The Effect of Biofeedback on Function in Patients with Heart Failure. Applied Psychophysiology and Biofeedback 34: 71–91. 19205870 10.1007/s10484-009-9077-2

[62] Cowan MJ, Pike KC, Budzynski HK (2001) Psychosocial Nursing Therapy Following Sudden Cardiac Arrest: Impact on Two-Year Survival. Nursing Research 50: 68–76. 11302295 10.1097/00006199-200103000-00002

[63] Bradley R, McCraty R, Atkinson M, Tomasino D, Daugherty A, et al (2010) Emotion Self-Regulation, Psychophysiological Coherence, and Test Anxiety: Results from an Experiment Using Electrophysiological Measures. Applied Psychophysiology and Biofeedback 35: 261–283. 20559707 10.1007/s10484-010-9134-x

[64] Kirschbaum C, Pirke KM, Hellhammer DH (1993) The ‘Trier Social Stress Test’ – A Tool for Investigating Psychobiological Stress Responses in a Laboratory Setting. Neuropsychobiology 28: 76–81. 8255414 10.1159/000119004

[65] Shirotsuki K, Izawa S, Sugaya N, Yamada KC, Ogawa N, et al (2009) Salivary cortisol and DHEA reactivity to psychosocial stress in socially anxious males. International Journal of Psychophysiology 72: 198–203. 19141305 10.1016/j.ijpsycho.2008.12.010

[66] Williams RA, Hagerty BM, Brooks G (2004) Trier Social Stress Test: A Method for Use in Nursing Research. Nursing Research 53: 277–280. 15266167 10.1097/00006199-200407000-00011

[67] de Timary P, Roy E, Luminet O, Fillée C, Mikolajczak M (2008) Relationship between alexithymia, alexithymia factors and salivary cortisol in men exposed to a social stress test. Psychoneuroendocrinology 33: 1160–1164. 18674866 10.1016/j.psyneuen.2008.06.005

[68] Edwards SL, Rapee RM, Franklin J (2003) Postevent Rumination and Recall Bias for a Social Performance Event in High and Low Socially Anxious Individuals. Cognitive Therapy and Research 27: 603–617.

[69] Mauss IB, Wilhelm FH, Gross JJ (2003) Autonomic recovery and habituation in social anxiety. Psychophysiology 40: 648–653. 14570172 10.1111/1469-8986.00066

[70] Weippert M, Kumar M, Kreuzfeld S, Arndt D, Rieger A, et al (2010) Comparison of three mobile devices for measuring R-R intervals and heart rate variability: Polar S810i, Suunto t6 and an ambulatory ECG system. European Journal of Applied Physiology 109: 779–786. 20225081 10.1007/s00421-010-1415-9

[71] Quintana D, Heathers J, Kemp A (2012) On the validity of using the Polar RS800 heart rate monitor for heart rate variability research. Eur J Appl Physiol. 10.1007/s00421-012-2453-2 22790488

[72] Russoniello C, Pougtachev V, Zhirnov E, Mahar M (2010) A Measurement of Electrocardiography and Photoplethesmography in Obese Children. Applied Psychophysiology and Biofeedback 35: 257–259. 20552266 10.1007/s10484-010-9136-8

[73] Hagströmer M, Oja P, Sjöström M (2006) The International Physical Activity Questionnaire (IPAQ): a study of concurrent and construct validity. Public Health Nutrition 9: 755–762. 16925881 10.1079/phn2005898

[74] Spielberger CD, Gorssuch RL, Lushene PR, Vagg PR, Jacobs GA (1983) Manual for the State-Trait Anxiety Inventory. Palo Alto, CA: Consulting Psychologists Press.

[75] Corruble E, Durrbach A, Charpentier B, Lang P, Amidi S, et al (2010) Progressive Increase of Anxiety and Depression in Patients Waiting for a Kidney Transplantation. Behavioral Medicine 36: 32–36. 20185399 10.1080/08964280903521339

[76] Hishinuma ES, Miyamoto RH, Nishimura ST, Nahulu LB, Andrade NN, et al (2000) Psychometric Properties of the State-Trait Anxiety Inventory for Asian/Pacific-Islander Adolescents. Assessment 7: 17–36. 10668003 10.1177/107319110000700102

[77] Millar K, Jelicic M, Bonke B, Asbury AJ (1995) Assessment of preoperative anxiety: comparison of measures in patients awaiting surgery for breast cancer. British Journal of Anaesthesia 74: 180–183. 7696068 10.1093/bja/74.2.180

[78] Hedberg AG (1972) State-Trait Anxiety Inventory. Professional Psychology Fall 3: 389–390.

[79] Boulez P (1957) Le Marteau sans maître. London: Universal Edition.

[80] Messiaen O (1942) Quatuor pour la fin du temps [music] : violon, clarinette en si bémol, violoncelle, et piano. Paris: Durand.

[81] Healy MJR (2000) Circadian rhythm of heart rate and heart rate variability - Commentary. Archives of Disease in Childhood 83: 182–182. 10.1136/adc.83.2.179 PMC1718415 10906034

[82] Tabachnick BG, Fidell LS (2007) Using multivariate statistics. Boston Pearson/Allyn & Bacon. 980 p.

[83] Hynynen E, Vesterinen V, Rusko H, Nummela A (2010) Effects of moderate and heavy endurance exercise on nocturnal HRV. Int J Sports Med 31: 428–432. 20419617 10.1055/s-0030-1249625

[84] Spielberger CD, Gorssuch RL, Lushene PR, Vagg PR, Jacobs GA (1983) Manual for the State-Trait Anxiety Inventory. Palo Alto, CA: Consulting Psychologists Press.

[85] Mizuno K, Tanaka M, Yamaguti K, Kajimoto O, Kuratsune H, et al (2011) Mental fatigue caused by prolonged cognitive load associated with sympathetic hyperactivity. Behav Brain Funct 7: 17. 21605411 10.1186/1744-9081-7-17 PMC3113724

[86] Goldstein DS, Bentho O, Park M-Y, Sharabi Y (2011) Low-frequency power of heart rate variability is not a measure of cardiac sympathetic tone but may be a measure of modulation of cardiac autonomic outflows by baroreflexes. Experimental Physiology 96: 1255–1261. 21890520 10.1113/expphysiol.2010.056259 PMC3224799

[87] Heathers JAJ (2012) Sympathovagal balance from heart rate variability: an obituary. Experimental Physiology 97: 556–556. 22525665 10.1113/expphysiol.2011.063867

[88] Pagani M, Lombardi F, Guzzetti S, Rimoldi O, Furlan R, et al (1986) Power Spectral Analysis of Heart Rate and Arterial Pressure Variabilities as a Marker of Sympatho-Vagal Interaction in Man and Conscious Dog. Circulation Research 59(2): 178–193. 2874900 10.1161/01.res.59.2.178

[89] Schulz K, Altman D, Moher D (2010) Group tC (2010) CONSORT 2010 Statement: updated guidelines for reporting parallel group randomised trials. Trials 11: 32. 21350618 10.4103/0976-500X.72352 PMC3043330

